# Need of the Hour: Family Medicine in India

**DOI:** 10.7759/cureus.24596

**Published:** 2022-04-29

**Authors:** Gokul Paidi, Anju Beesetty, Abdelilah Lahmar, Lisa Kop, Ranbir Sandhu

**Affiliations:** 1 Family Medicine, The Medina Clinic, Grandview, USA; 2 Obstetrics and Gynecology, Southern Medical University, Guangzhou, CHN; 3 Medicine, Faculty of Medicine and Pharmacy, Mohammed VI University Hospital, Oujda, MAR; 4 Neurology, Zaporizhzhia State Medical University, Zaporizhia, UKR; 5 Urgent Care, San Luis Walk In Clinic, Inc., Somerton, USA

**Keywords:** mbbs syllabus, medical education in india, mbbs, diplomate, integrated approach, primary care, family medicine

## Abstract

Family medicine practitioners can manage a wide range of health problems, either directly or through referral to other specialists, thereby optimizing the cost-effectiveness of the healthcare system. For most health problems, family medicine practitioners serve as an entry point for individualized healthcare. Providing comprehensive care for most specialties for all ages is the hallmark of family medicine. It is recognized as a distinct specialty in many developed countries, and training is provided through organized residency programs. Family medicine has been incorporated in the foundation course of Bachelor of Medicine, Bachelor of Surgery (MBBS) and is planned to be adopted in all medical institutes in India; however, the MBBS syllabus includes no mention of it. In medical schools in India, fully functioning family medicine departments should be established on an urgent scale. It is necessary to take the required measures to introduce the subject into the undergraduate MBBS program and to generalize it across India. It is recommended that the number of medical schools offering postgraduate residency training in family medicine be increased to meet current community health needs.

## Introduction and background

Family medicine is a specialty that aims to provide comprehensive care to individuals and families. This includes diagnosis and treatment or referral, chronic and long-term care, palliative care, health promotion, and disease prevention activities. It encompasses a spectrum of care and services to meet the entire needs of the community. A family medicine practitioner provides primary and secondary healthcare to all patients, regardless of age, gender, or health problems. The family medicine practitioner can address a wide range of health issues, either through direct care or through referral to other specialties, thereby maximizing the cost-effectiveness of the healthcare system [1].

Family doctors provide personalized healthcare services for most health problems and act as the first line of contact with the healthcare system. Quality primary care has been associated with diagnostic accuracy, a reduction in avoidable hospitalizations, and better health outcomes [2]. A well-trained family health practitioner who can provide integrated healthcare is urgently needed to improve the current healthcare system in India. A Bachelor of Medicine, Bachelor of Surgery (MBBS) graduate who has completed the MBBS course and is registered with the Medical Council of India (MCI) is considered a general practitioner. Even if such doctors acquire a wealth of knowledge through years of practice, they do not go through any special training. This can affect the quality of the healthcare they provide [3].

A general practitioner, on the other hand, receives specialized training in the field of family medicine. Due to the increasing demand for specializations in other core areas by MBBS graduates, interest in family medicine is gradually declining. The importance of a family physician providing comprehensive care both in the hospital and in the community is often underestimated. The scope of family medicine is broad and includes care at various stages such as pregnancy, newborn care, child care, treatment of acute and chronic adult life diseases, and end-of-life care. The transition to universal health coverage for all requires a change in the healthcare system that places more emphasis on the practice of family medicine [4,5]. 

## Review

History of family medicine in India 

**Figure 1:**
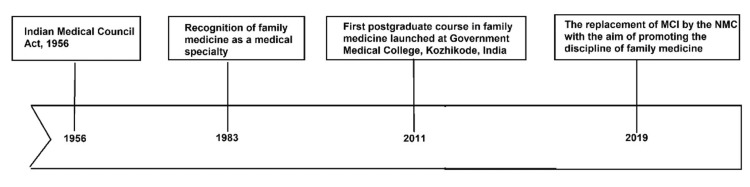
The figure shows the chronological history of family medicine in India MCI: Medical Council of India; NMC: National Medical Commission Image credits: Abdelilah Lahmar

As a new area of specialization, family medicine first emerged in western countries as a need for personal healthcare. Family health practitioners play different roles that vary from country to country. Many countries recognized the importance of family medicine in improving healthcare systems and introduced family medicine as a separate specialty into their health systems [6]. The concept of family medicine has evolved mainly to meet people's growing health needs. In India, the first postgraduate course in family medicine was launched in 2011 at the Government Medical College in Kozhikode, Kerala [7]. Family medicine was originally recognized as a medical specialty in India in 1983 when the MCI Act 1956 was amended [8]. A three-year residency program in Family Medicine is required by the MCI to award the Diplomate of National Board (DNB) or the Doctor of Medicine (MD) in Family Medicine [6]. The primary objective of a postgraduate course in family medicine is to educate specialists in family medicine to provide quality healthcare to the community and advance the medical course through research and education. As of 2018, 10 MD family medicine postgraduate residency training positions and 200 DNB postgraduate positions are available in India each year [6]. In many developed countries, family medicine is recognized as a distinct specialty, and training is provided through structured postgraduate residency programs [6-8]. However, awareness of the specialty of family medicine is low among undergraduate students in India [9,10]. Interestingly, the Indian Parliament has passed the National Medical Commission (NMC) Act in 2019. The NMC replaces the MCI Act and aims to facilitate the delivery of quality primary care as well as promote family medicine as a discipline [11]. In addition, family medicine has been included in the foundation course of MBBS and is set to be introduced in all medical institutions in India, although the current MBBS syllabus makes no mention of it [11]. Figure 1 shows the chronological history of family medicine in India

Current challenges to the health system in India

The current healthcare system in India is very complex, ranging from well-equipped and advanced private hospitals with specialist doctors to inadequate public health services at the primary care level [12]. Although healthcare resources are growing, the healthcare system remains largely underserved and unequally distributed [12]. In India, the majority of the population resides in rural areas that are poor and generally underserved in terms of health care [12]. On the other hand, privatized healthcare system in India is expensive and urban-oriented. Even among the urban population, there is a large disparity; for example, rich individuals are admitted more often than poor people and have longer inpatient stays in public hospitals [12]. In most Indian cities, patients rely on tertiary care hospitals due to a lack of comprehensive health care even for minor illnesses. The cost of providing specialty care far outweighs the cost of primary care. In resource-poor countries such as India, there already exists a shortage of physicians and healthcare infrastructure across the board; the absence of family medicine physicians further exacerbates the healthcare gap. By implementing a robust network of family medicine practices, efficient primordial and primary care could be delivered at low costs and thus prevent the need for specialty care, which is often more expensive. The tradition of the family doctor is gradually declining in India due to the increasing demand for specialization in medicine and lack of knowledge about family medicine. One can conceptualize these challenges that the Indian healthcare system faces into two entities: the healthcare supply challenge and the demand for healthcare challenge.

Challenges Related to Supply of Healthcare

The imbalance in allocation contributes negatively to ensuring the availability of adequate and well-resourced health services. Although low public funding and significant interstate disparities exacerbate the problem, a larger proportion of resources are allocated to urban healthcare services compared to rural healthcare services [12]. Physical access to preventive and curative healthcare is a key barrier for India's rural population. Moreover, public hospital beds in cities are more than double those in rural areas, and the emergence of the private sector in cities has led to an unplanned and uneven geographic distribution of services [12,13]. A 2012 survey in India found that only 37% of rural people could use inpatient facilities within a 5 km radius, while 68% had access to outpatient services [14]. In another study, it was postulated that the more rural on's location is, the more out of the city one lives, the greater the risk of disease, malnutrition, frailty, and early mortality [14]. Furthermore, even when services are accessible, physical access to them does not guarantee their use, as the cost of seeking care can deter its use [14]. According to a study of six Indian states, many primary health clinics lacked basic infrastructures such as beds, wards, bathrooms, drinking water facilities, clean delivery rooms, and consistent electrical supply [14]. 

Medical staff absenteeism, with its sub-optimal distribution, is a key element in affecting the quality of medical care provided to patients. According to a 2011 survey, India has about 20 health workers per 10,000 population, with allopathic doctors making up 31% of the workforce, nurses and midwives 30%, and pharmacists 11%. [15]. In addition, the workforce is not evenly distributed, with the majority choosing to work in places with better infrastructure and opportunities for family life and advancement [14]. Thre is also the existence of underqualified private and government providers, a point previously identified in a study conducted in rural Rajasthan, which found that 40% of providers had no medical degree and almost 20% had no secondary education [12]. 

Challenges Related to Demand for Healthcare

According to data from national expenditure surveys, inequalities in health financing have worsened over the past two decades [12]. Only about 10% of India's population is covered by social or voluntary health insurance, mostly provided through government plans for certain categories of organized sector workers [12]. Poor individuals are more sensitive to healthcare costs and more likely to delay treatment when ill, and this impact has increased over time for residents of rural and metropolitan areas [12]. In addition, the financial burden of inpatient and outpatient treatment for rural families is consistently higher than for urban households, with significantly increasing expenditures per admission [12]. As a result, higher out-of-pocket health spending exacerbates poverty. Corruption, knowledge, education, and information can affect health beliefs, perceptions of health and illness, health-seeking behavior, and adherence to therapy, which impacts proper demand for and adherence to health services [12]. 

**Figure 2:**
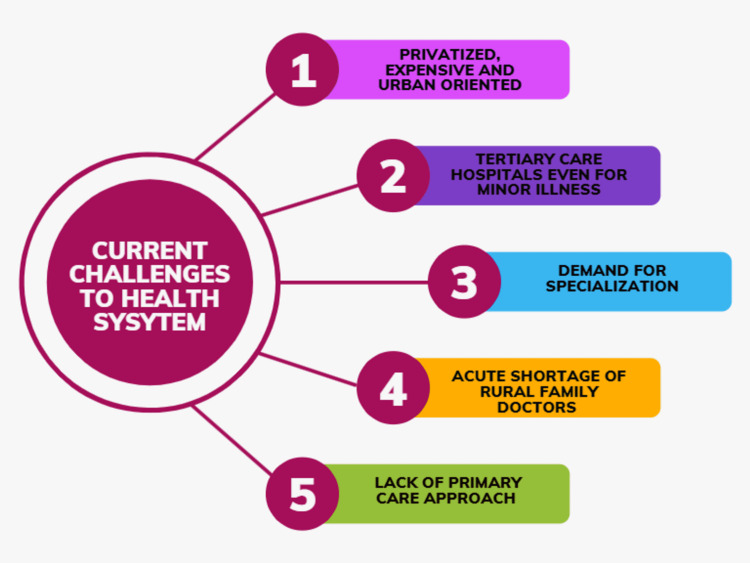
Current challenges in healthcare system Image Credits: Gokul Paidi

The primary care approach focuses more on health promotion and disease prevention than on treating the disease. The difficulty of providing the rural areas of India with adequate medical care is mainly due to the acute shortage of rural medical practitioners. Providing an efficient and effective healthcare system requires well-trained general practitioners who practice evidence-based medicine with locally relevant expertise [9]. Figure 2 describes current challenges in the healthcare system in India.

Patient-centered approach versus disease-centered approach

**Figure 3:**
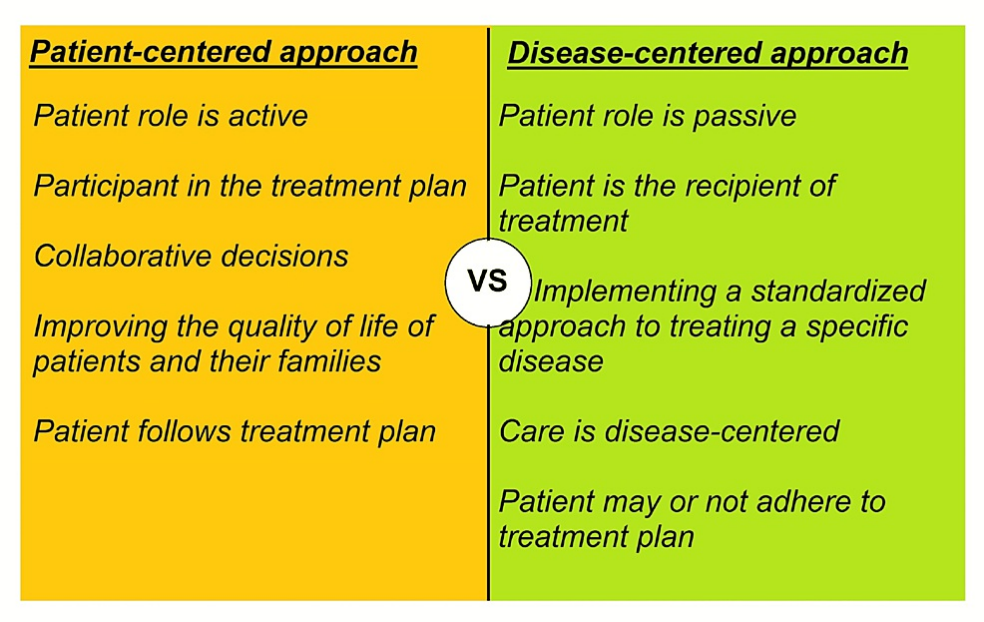
The difference between the patient-centered approach and the disease-centered approach Image credits: Abdelilah Lahmar

The practice of family medicine is largely preventative care and the overall well-being of the patient is the foremost priority of the family physician. Therefore it can be argued that family medicine employs a patient-centered approach rather than a systems- and disease-based approach as seen in specialty care. The family doctor is a personal doctor who provides primary care and also coordinates the care if necessary by referring to other specialists and not just focusing on disease management [10]. A family doctor also improves the quality of life for the patients and families. Family medicine provides safe and effective care for all family members [10]. In the absence of good family medicine practice in a community, there is an increased incidence of lifestyle-related diseases and patients often present in advanced stages of illness. Due to the complexity of care in an advanced illness, patients often feel challenged and they are not able to fully participate in their healthcare decisions, thus, leading to a disease-centered, paternalistic care. Family medicine physicians are also strong health educators and they can help mitigate the gap between patients and healthcare providers, thus empowering patients to take part in their healthcare. Patient-centered care takes into account the specific needs of patients when treating diseases and places more emphasis on participatory medicine by involving patients in their health decisions and choices. In patient-centered care, the patient's needs, preferences, and values are considered in the clinical decision-making process. Figure 3 shows the difference between the patient-centered approach and the disease-centered approach 

Family medicine practitioner: role and responsibilities

Family medicine is a community-based and integrated approach that includes case management, long-term care, and care coordination to provide quality healthcare to people [16]. It can address the majority of the health needs of the population. A family medicine physician should provide both primary and secondary levels of health care to all family members and be the first line of contact with the family for health care [17,18]. Some of the specific roles and responsibilities of a family physician are: 1) Promote health and prevent disease in the family and community; 2) Manage common diseases in all age groups across different clinical specialties and utilize the limited resources in primary care; 3) Appropriate referral to specialists by correctly identifying the red flags and symptoms; 4) Provide continuity of care by involving in two-way referral with the specialists; 5) Provide home and palliative care; 6) Provide community-focused care with a multidisciplinary approach; 7) Should be aware of the associated socio-cultural and environmental issues related to health; and 8) Provide effective health education to the family and the community. 

**Figure 4:**
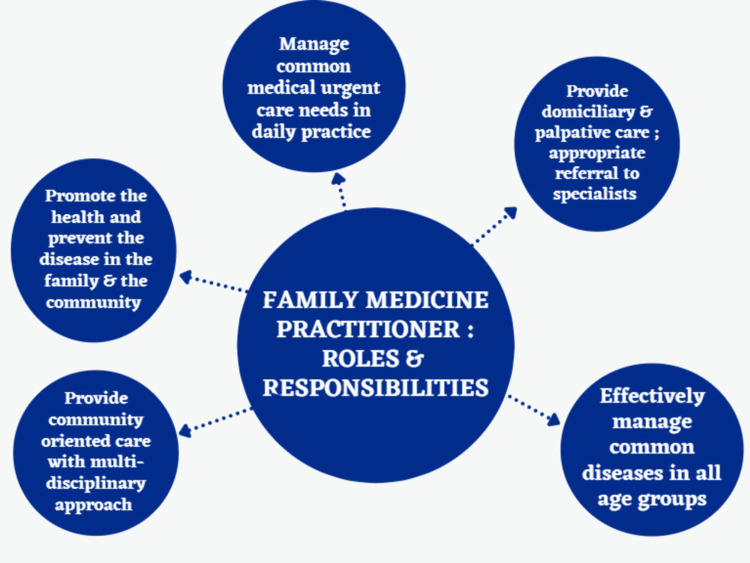
Family medicine practitioner: role and responsibilities Image credits: Gokul Paidi

Family medicine is based on continuity of care and creates a long-term relationship between patients and doctors. Chronic conditions such as asthma, hypertension, and diabetes can be actively managed in primary care, reducing hospitalizations and preventing acute exacerbations of these conditions. Continuity of care has been linked to improved patient satisfaction, lower healthcare costs, and accountability [19]. Family medicine physicians often have a longer therapeutic relationship with the patients and often are more trusted by patients than other healthcare providers because of the longitudinal relationship. By virtue of this trustful physician-patient relationship, family medicine doctors can be strong advocates for their patients and they can facilitate communication in a medical team and offer a co-production of healthcare here information and decision-making are shared [20-22]. Family practitioners also coordinate service delivery across different healthcare services. By coordinating care, the approach brings together healthcare professionals and providers to ensure the patient receives appropriate care in diverse settings. It brings together professionals from different disciplines to work in partnership to ensure collaborative care. Patients' perceived ability to access health services will be more in terms of timeliness and ease of access [23,24]. Family medicine also empowers people and the community to take control of their own health, leading to healthy behaviors. Figure 4 explains the roles and responsibilities of a family practitioner.

The way forward

Departments for family medicine should be set up in medical colleges all over India. The necessary steps should be taken to incorporate the family medicine curriculum into the undergraduate MBBS curriculum. The number of medical colleges offering postgraduate residency training in family medicine should be increased. Public healthcare institutions and organizations should be educated regarding family medicine. A comprehensive curriculum for family medicine needs to be developed to meet the current health needs of the community. The health system should focus more on primary care and family medicine to provide people with affordable, accessible, and quality healthcare. A primary care-based health system is required for the epidemiological and nutritional transition as well as the impact of urbanization on health. In order to improve rural health services in India, sufficient numbers of rural family doctors are also needed, which can be achieved by improving and expanding family medicine specialty in India. 

## Conclusions

India is experiencing a rapid increase in population every year and the majority of the population lives in rural locations. Access to affordable, quality healthcare in rural India has been a challenge. Family medicine practitioners can play a big role in providing easily accessible, low-cost, high-quality healthcare to not just rural populations but also in urban centres where the wait time for specialists may be very high. However, family medicine is still not considered a specialty in India, and awareness about family medicine among the public and health stakeholders is low. Family medicine is evidence-based, patient-centered, community-focused, and problem-focused. A family medicine physician also improves the economics of healthcare by treating the most basic ailments of diseases across most specialties and then referring them to appropriate specialists through triage and initial treatment. Family medicine takes a collaborative approach that aims to increase the health and well-being of the family by focusing on individual needs and preferences. Family medicine practitioners work interdisciplinary with patients and play a crucial role at the interface of many specialties in the healthcare system. The increasing trend toward specialization in medicine, combined with the provision of a decentralized approach to healthcare, increases the need for family medicine in the country as it cuts the traditional boundaries of specialties in medicine to provide quality healthcare.
